# Multi‐Scale Engineering of Photonic Meta‐Structures With Emergent Optical Resonances via Colloidal Crystal Engineering With DNA and Microwell Templates

**DOI:** 10.1002/smll.75174

**Published:** 2026-08-12

**Authors:** Alexa M. Wong, Ramin Yazdaanpanah, Mia J. P. Pascall, Jeongmin Cho, Yiming Yang, Allen X. Guo, Janice Kang, Koray Aydin, Chad A. Mirkin

**Affiliations:** ^1^ Department of Chemistry Northwestern University Evanston Illinois USA; ^2^ International Institute for Nanotechnology Northwestern University Evanston Illinois USA; ^3^ Department of Electrical and Computer Engineering Northwestern University Evanston Illinois USA; ^4^ Department of Materials Science and Engineering Northwestern University Evanston Illinois USA

**Keywords:** colloidal crystals, DNA, metamaterials, nanoparticles, resonator

## Abstract

Metasurfaces offer control over light–matter interactions, but their material scope is often constrained by top‐down fabrication techniques, limiting compositional flexibility in meta‐atoms and the accessible optical properties. Herein, a bottom‐up approach integrates colloidal crystal engineering with DNA with lithographically defined microwell templates to create meta‐structures composed of nanoparticle superlattices that function as programmable meta‐atoms. The microwell environment stabilizes non‐equilibrium superlattice habits inaccessible with homogeneous crystallization, decoupling habit formation from lattice symmetry. This approach enables tuning of optical response through hierarchical parameters: 1) nanoparticle composition and size, DNA length, and lattice symmetry define superlattice optical characteristics; 2) superlattice size and habit determine resonator supported modes; and 3) inter‐superlattice spacing and arrangement govern collective lattice effects. This multiscale modularity enables access to a vast combinatorial design space for optical engineering. As proof‐of‐concept, a meta‐structure comprised of periodic cylindrical superlattices exhibiting a near‐infrared Mie‐ resonance is fabricated. Despite being composed of metallic gold nanoparticles, the superlattices exhibit an emergent dielectric‐like response with low effective extinction in the NIR/mid‐IR regime, enabling volumetric electromagnetic field penetration. Field maps show resonant behavior characteristic of magnetic dipole Mie modes. By integrating colloidal assembly with system‐level optical functionality, this work establishes a bottom‐up framework for designing photonic meta‐structures.

## Graphical abstract

This work presents a bottom‐up strategy for forming meta‐structures built from DNA‐assembled nanoparticle superlattices, establishing a versatile platform for multiscale photonic materials design. Confinement stabilizes non‐equilibrium crystal habits and enables geometry and mode control independent of lattice symmetry. Electromagnetic simulations indicate the resulting arrays exhibit an emergent dielectric‐like response with low effective extinction despite using gold nanoparticles, making the superlattices programmable metamaterial meta‐atoms.

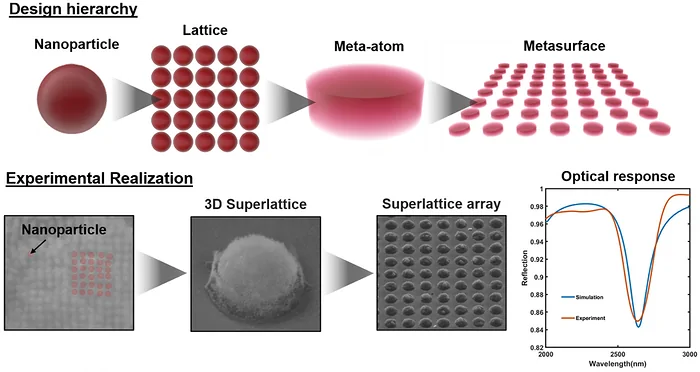

## 1 Introduction

Colloidal nanoparticles (NPs) are versatile building blocks for the bottom‐up assembly of micro and macroscale materials and devices [1, 2]. Their tunable size, shape, and surface chemistry [3, 4] provide a robust toolkit for constructing three‐dimensional (3D) superlattices, or colloidal crystals. Among the ligands used to guide NP assembly, DNA provides exceptional chemical and structural programmability, enabling sub‐nanometer architectural precision [5, 6, 7] in superlattices spanning over 100 distinct crystal symmetries to date [8, 9, 10]. In colloidal crystal engineering with DNA, NP cores are densely functionalized with radially oriented DNA shells to form ‘programmable atom equivalents’ (PAEs, synthetic analogues of atoms whose valencies and binding specificities are encoded by number of oligonucleotides, particle shape, and complementary base pairing interactions).

The complementary contact model (CCM) posits that in the most stable structures PAEs adopt configurations that maximize DNA hybridization events [7, 11]. This principle offers a predictive framework linking PAE geometry, DNA loading, and sequence complementarity to directional bonding and the resulting symmetry of the NP lattice. PAE superlattices exhibit properties different from those of the individual constituents, including tunable plasmon coupling [12], multipolar Mie resonances [13], and second harmonic generation [14]. Despite the high degree of structural control achievable with colloidal crystal engineering with DNA, the range of crystal habits accessible using this strategy alone remains limited. This constraint leaves a critical design space unexplored, but rich with potential, as superlattice habit is known to strongly influence the optical responses of colloidal crystals [13, 15, 16, 17].

In colloidal crystal engineering with DNA, experimental conditions dictate whether crystallization is thermodynamically governed, yielding Wulff shapes [18, 19], or kinetically dominated, often resulting in amorphous architectures [20]. Indeed, equilibrium‐driven colloidal crystallization favors habits enclosed by the lowest‐energy crystal planes, limiting the possible habits that are accessible. Alternatively, non‐equilibrium habits can form under specific conditions in kinetically driven crystallization regimes. For example, the non‐equilibrium habits of CsCl [21] and AB [22] superlattices can be attained by modulating the temperature (i.e., cooling rates) of solutions of PAEs as they crystallize. In addition, colloidal crystals with polydisperse habit sizes form due to stochastic nucleation events and local concentration fluctuations [23, 24, 25]. Ultimately, the stochastic nature of crystallization has made it difficult for researchers to deliberately target specific crystal habit shapes and sizes. As a result, tuning the optical properties that depend on those parameters remains challenging under most experimental conditions explored thus far.

Lithographically defined microwells were recently introduced as a strategy to enable controlled heterogeneous nucleation during colloidal crystallization [26, 27, 28]. With this approach, a resist‐coated, gold‐coated silicon wafer is patterned by e‐beam lithography to generate uniform microwells, where the exposed gold surface at the bottom of each well can be functionalized with DNA to direct the site‐specific crystallization of PAEs during slow cooling. This approach effectively controls superlattice placement, in‐plane size, orientation, and dispersity. Herein, this approach is leveraged to investigate colloidal crystallization pathways as a function of supersaturation in the presence of the microwells. The confined microwell environments stabilize non‐equilibrium habits by delaying superlattice faceting. This strategy permits the use of microwell geometry to reproducibly generate metastable superlattices with prescribed habits. Habit control is functionally significant, as the geometry of a superlattice dictates the optical mode it supports. As a proof‐of‐concept, this approach was utilized to create a structurally programmed optical device composed of an array of cylindrical metastable superlattices, each serving as a magnetic Mie resonator. Reflection measurements revealed a magnetic‐mie resonance at ≈2,600 nm. Importantly, this DNA‐based assembly strategy provides modular control over the structural parameters of colloidal crystals across nano‐ and mesoscales. Each of these parameters can be tuned independently to tailor the optical responses of hierarchical NP superlattice metasurfaces.

## 2 Results and Discussion

### 2.1 Supersaturation‐Dependent Crystallization Pathways

Heterogeneous nucleation processes lower the critical nucleation barrier by reducing surface energy through interfacial interactions. However, once critical nuclei form in solution, homogeneous nucleation and growth can proceed rapidly, competing with surface‐directed crystallization. Classical nucleation theory (CNT) suggests that the balance between these pathways can be modulated by supersaturation [29]. Formally, CNT defines supersaturation as the logarithmic ratio of concentration to equilibrium solubility. However, in colloidal assembly systems, similar to protein crystallization, equilibrium solubility is not a fixed or measurable quantity. Accordingly, we define supersaturation as an operational, regime‐based measure of concentration beyond equilibrium at a given time in the crystallization process. At low supersaturation, the high barrier for homogeneous nucleation suppresses bulk crystallization, allowing heterogeneous nucleation to dominate. In contrast, high supersaturation lowers the nucleation barrier for both processes, enabling spontaneous nucleation in solution. To explore these crystallization processes in the context of a PAE system, equal quantities of PAEs were solvated in different volumes of 0.5 M NaCl buffer to systematically vary the concentration to target different supersaturation regimes (as determined using the optical density (OD) of the solution at 520 nm (OD at 520 nm = 2.4, 5, and 10). The microwell count on the substrates was kept constant at n = 5,000. After slow‐cooling, visual inspection of the solutions revealed increasing amounts of solid products at the bottom of the tubes as supersaturation increased (Figure 1A–C). To determine whether this observation correlated with changes in surface‐based crystallization, the substrates were analyzed using scanning electron microscopy (SEM) after silica encapsulation, performed using a modified protocol [30] designed to prevent silica infiltration into the PMMA matrix, a common issue with prior methods that led to irreversible silica deposition, making the PMMA resistant to removal methods (Figure S1, see Methods for details).

**FIGURE 1:**
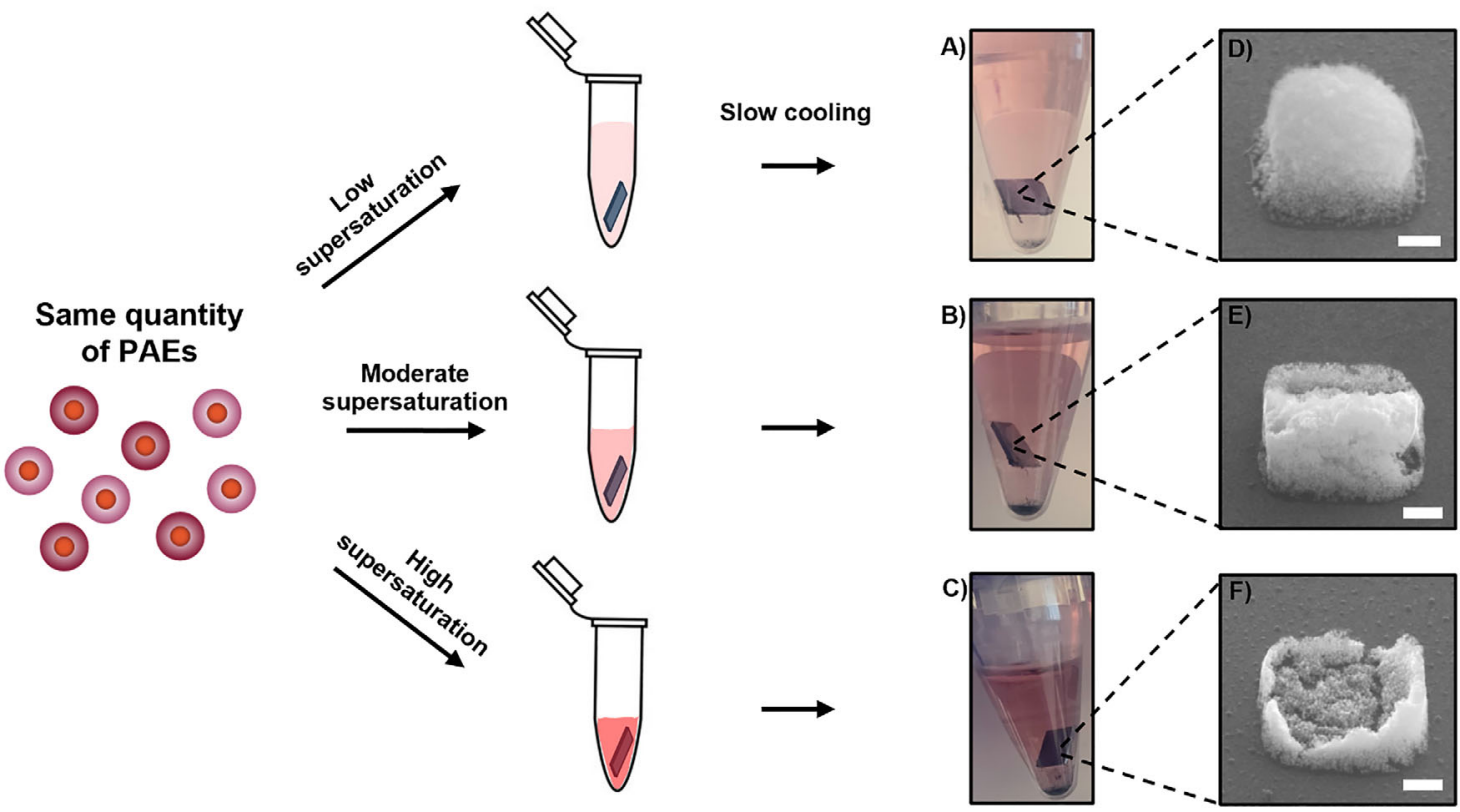
Supersaturation‐dependent crystallization pathways. (A–C) Photographs of PAE solutions after a slow‐cooling protocol under different supersaturation conditions. (A) At low supersaturation (total volume = 2500 µL, 2.4 OD), no significant solution‐based products were observed. (B,C) At moderate and high supersaturation (total volume = 1200 µL and 600 µL, 5 and 10 OD, respectively), visible solution‐based products accumulate at the bottom of the tubes. (D‐F) Tilt‐view (45˚) SEM images of superlattices collected from the substrate. (D) At low supersaturation and high PAE loading in the microwells, intact superlattices are observed. (E,F) At high supersaturation, PAE loading in the microwell is decreased and the superlattices are irregular and fragmented. Scale bars: 200 nm.

A tilt stage was used during SEM imaging to visualize the 3D architecture of the superlattices as top‐down views alone do not capture the extent of particle accumulation within the microwells. SEM imaging revealed that microwell‐assembled superlattices were significantly larger and more well‐formed when low supersaturation solutions (Figure 1D) were used compared to when moderate and high supersaturation conditions were used (Figure 1E,F). Additional SEM images are shown in Figures S2–S4. These results support an inverse relationship between heterogeneous nucleation and supersaturation level (Figure 2); at low supersaturation, heterogeneous nucleation (surface‐based) processes involve a larger fraction of material, and at high supersaturation, heterogeneous crystallization involves a lower fraction of the total material. This result shows that nucleation pathways can be modulated by toggling experimental conditions.

**FIGURE 2:**
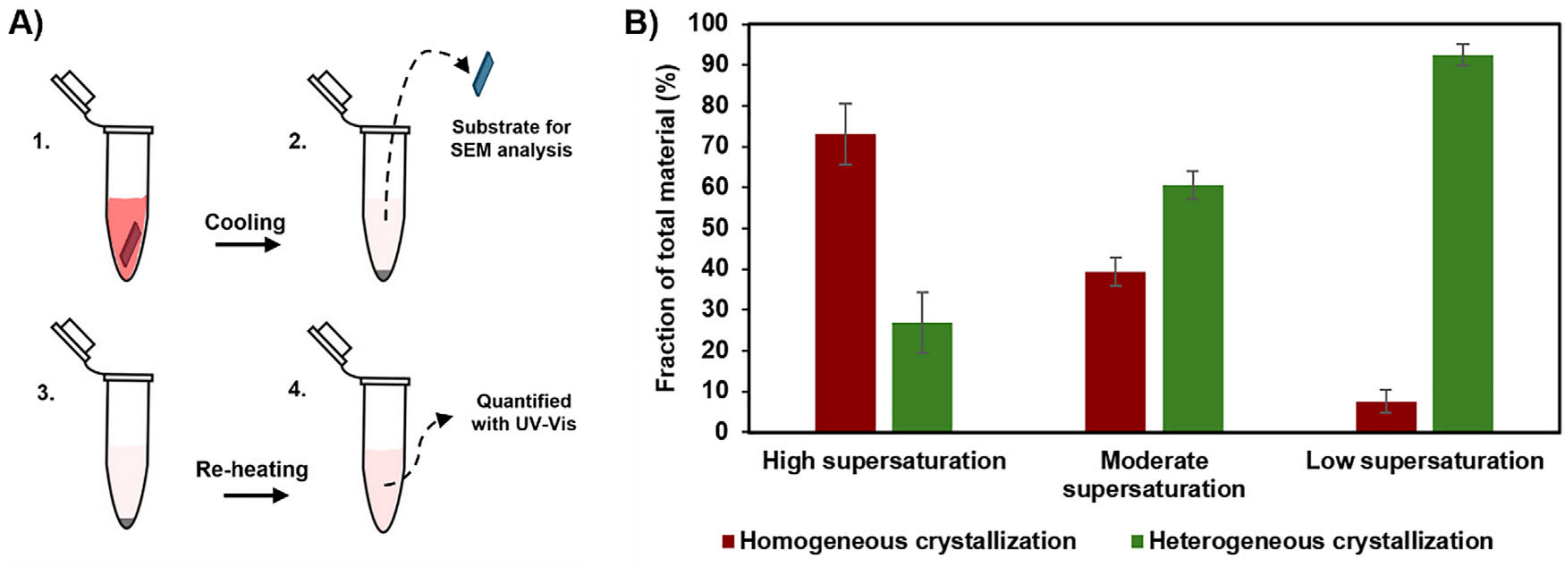
(A) Schematic of the experimental workflow. (B) PAE distribution between homogeneous (red) and heterogeneous (green) pathways at varying supersaturation levels.

### 2.2 Quantifying the Partitioning of PAEs Between Homogeneous and Heterogeneous Crystallization Pathways

Following the crystallization protocol with each type of solution, we quantified the fraction of PAEs crystallized in solution (and therefore involved in homogeneous pathways) along with the unreacted PAEs (Figure 2A). To do so, the supernatant and any solution‐based assemblies were reheated above the melting temperature of the PAEs to dissolve all thermally reversible crystalline products. The PAE concentration in this solution was then quantified using UV‐visible spectroscopy by measuring absorption at 520 nm (the extinction peak of the 20‐nm AuNPs) (Figures S5–S7). We assumed that the remaining fraction of PAEs were incorporated into the substrate‐bound assemblies. Thus, this approach served as an indirect, quantitative estimate of heterogeneous (on the substrate) vs. homogeneous (in solution) crystallization. Indeed, the results (Figure 2B) reveal an inverse correlation between homogeneous and heterogeneous nucleation, dependent on supersaturation levels. At high supersaturation, approximately 75% of the total material was recovered in the supernatant, while ≈25% contributed to substrate growth. At moderate supersaturation, substrate growth increased to over 60% with a corresponding drop in solution‐based crystallization to ≈40%. Notably, at low supersaturation, over 90% of the total material assembled on the substrate, and less than 10% formed solution‐based products. These results corroborate the hypothesis that reducing supersaturation biases nucleation toward heterogeneous crystallization pathways, offering a route to improved control in superlattice fabrication on substrates.

While this trend is consistent with the thermodynamic rules underpinning classical nucleation theory, where the free energy barrier for heterogeneous nucleation is lower [31], kinetic considerations likely play an important role as well. In low supersaturation systems, the reduced collision frequency of PAEs suppresses homogeneous nucleation by lowering the probability of critical nuclei formation in solution [32, 33, 34]. In contrast, surfaces can stabilize subcritical clusters by lowering interfacial energy and reducing the entropic cost of ordering [31]. These kinetic and thermodynamic advantages enable heterogeneous nucleation to proceed more readily than homogeneous nucleation under low supersaturation conditions.

### 2.3 Superlattice Habit Evolution Within Microwells Under Low Supersaturation Conditions

Microwell templates can be used to spatially confine nucleation to predefined sites, yielding one superlattice per well and enabling reliable control over crystal number and location. Combined with the strong preference for heterogeneous nucleation under low supersaturation, spatial confinement enables more reproducible superlattice formation by directing PAEs toward predefined nucleation sites. For instance, increasing the quantity of PAEs in solution should primarily fuel the growth of a finite number of superlattices (i.e., one per microwell), rather than generating new assemblies in solution. To test this hypothesis, the quantity of PAEs in solution was systematically varied while microwell count remained constant at 400. All samples were subjected to the same slow‐cooling protocol under low supersaturation conditions. An increase in PAE quantity correlates with an increase in superlattice z‐height, crystallinity, and habit evolution (Figure 3B–D).

**FIGURE 3:**
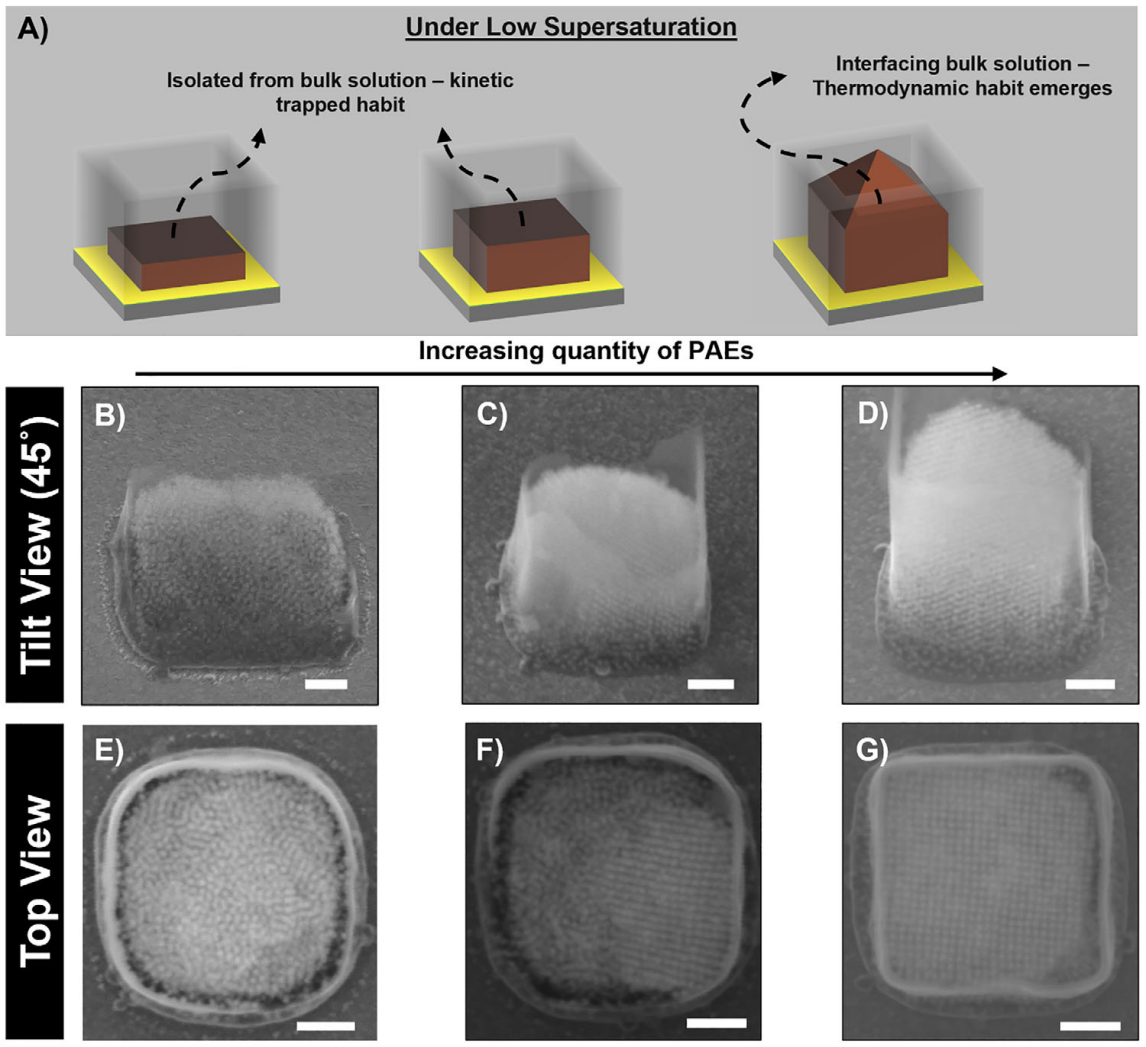
(A) Schematic of superlattice habit evolution within microwells under low supersaturation (total volume = 2,500 µL). (B–D) Tilt‐view (45˚) SEM images of superlattices with increasing z‐height. (E–G) Top‐down view SEM images of superlattices with increasing ordering. Superlattice obtained from a (B,E) 0.96 OD, (C,F) 1.2 OD, (D,G) 1.44 OD PAE solution. Scale bars: 200 nm.

At low loading (0.96 OD), superlattices remained deeply seated within the microwells and displayed a polycrystalline structure with short‐range order (Figure 3E). At intermediate loading (1.2 OD), superlattices grow to occupy a greater fraction of the microwell volume, and their crystallinity is improved; grain alignment can be observed (Figure 3F). At the highest PAE loading (1.44 OD), superlattices approached the top of the microwell, where a clear transition to a single‐crystalline habit was observed. The side‐view showed faceting consistent with the ideal BCC morphology (Figure 3D). The top‐view SEM revealed a continuous grain across the entire top facet (Figure 3G), indicative of a single‐crystalline domain. Superlattice size and quality were uniform throughout the arrays based on SEM observations (Figures S8–S10).

As the superlattices increased in height and approached the bulk solution, their crystallinity improved, and faceting occurred (Figure 3G). The observation that habit evolution initiates only as superlattices grow towards the upper boundaries of the microwells is consistent with prior reports that describe how physical confinement can preserve non‐equilibrium morphologies formed under kinetic‐driven growth conditions [35, 36, 37]. Since all assemblies underwent an identical slow‐cooling protocol, annealing time can be ruled out as the cause of this observation. In addition, even with extended annealing times, the superlattices confined within microwells remain kinetically trapped in non‐equilibrium habits (Figure S11). These results indicate that the non‐equilibrium habits are metastable and not transient kinetic intermediates and that the microwells are kinetically controlled environments that restrict the access of the crystals to thermodynamic minimum. This work suggests that a combination of geometric confinement and kinetically driven growth conditions, sustained by convective flow and continuous supply of building blocks, favors non‐equilibrium growth.

### 2.4 Metastable Superlattices with Habits by Design

Since non‐equilibrium superlattices did not exhibit faceting, we hypothesized that their overall morphology could be directed by the geometry of the microwell template. To explore this hypothesis, microwells with triangular prism, cylindrical, and ring‐like geometries were prepared for guided superlattice growth under low supersaturation conditions. Interestingly, the resulting superlattices closely conformed to the shapes of the microwell, producing uniform superlattice shaped like triangular prisms, cylinders, and rings (Figure 4A–C). High‐resolution SEM imaging confirmed that, although the internal ordering was short‐ranged, the superlattice habit was dictated by the microwell geometries (Figure 4D–F). These observations support the idea that, in the absence of a sufficient thermodynamic driving force to induce facet formation, the superlattice growth is driven predominantly by geometric (microwell dimensions) and kinetic factors. The successful synthesis of more complex superlattice habits, such as the ring‐like structure (Figure 4F), demonstrates the ability of the system to resolve fine‐templated features, despite nanoscale packing constraints. This kinetic programmability presents a powerful route to control superlattice habit independent of crystallographic symmetry.

**FIGURE 4:**
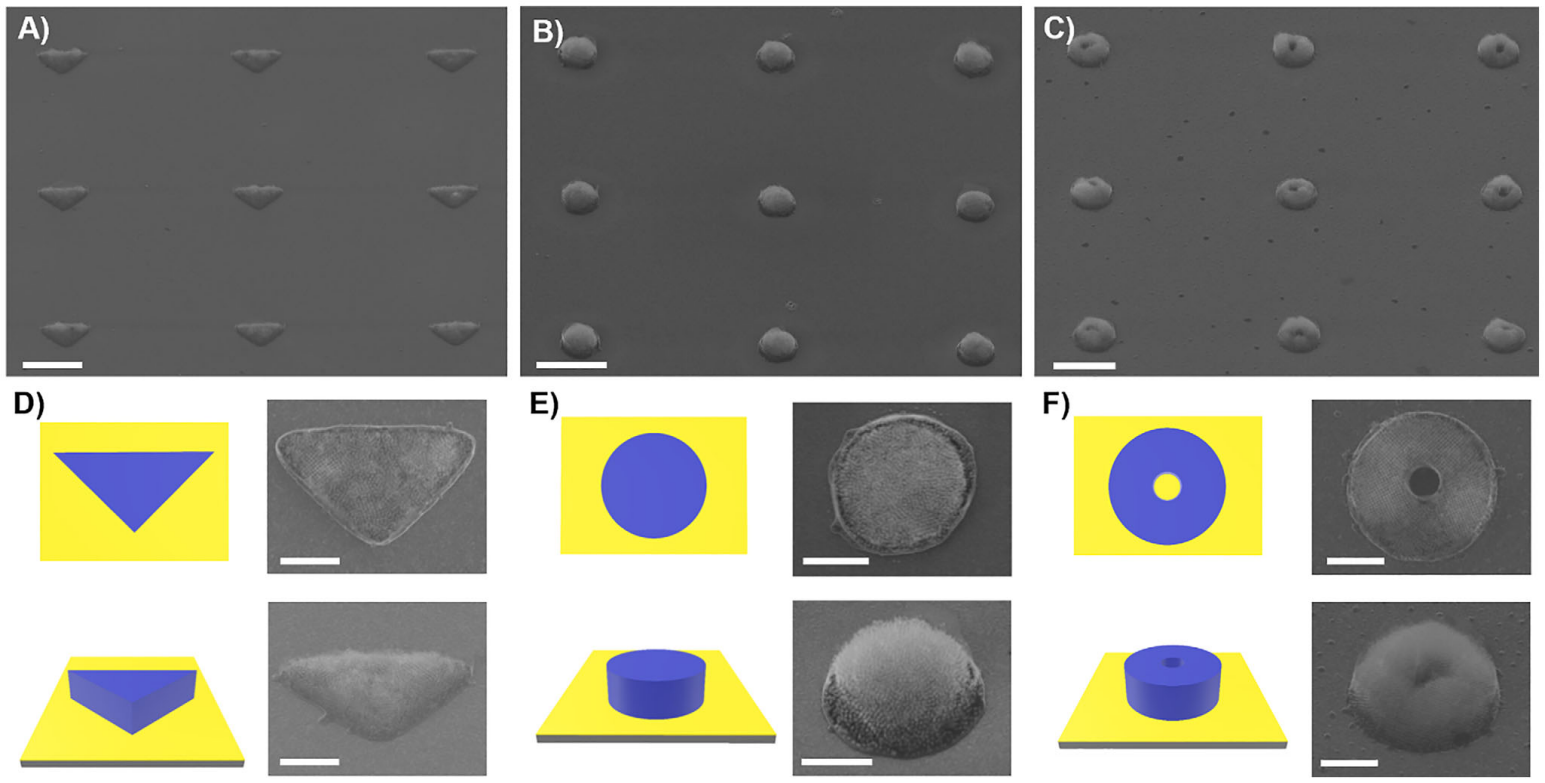
(A–C) SEM images showing arrays of superlattices grown in (A) triangular prism, (B) cylindrical, and (C) toroidal microwells. Scale bars: 2 µm. (D–F) Top rows: Schematic of each microwell geometry and corresponding top‐view SEM images of individual superlattices, revealing close conformity between superlattice footprint and well geometry. Bottom rows: Schematic 3D models of each microwell (left) and corresponding 45° tilt‐view SEM images of superlattices, showing superlattice habit in all three dimensions. Scale bars: 500 nm. All grown in 1.2 OD colloidal solutions.

Simulations were performed to investigate how the habits of these metastable superlattices affect their array level far‐field optical properties. First, using scattering matrix theory (see Supplemental Information), the real (n) and imaginary (k) parts of the effective refractive index for a perfect BCC lattice composed of 20‐nm AuNPs with a lattice parameter of 40 nm was calculated. The lattice parameter was chosen based on the expected interparticle spacing derived from the DNA linker length and NP size and is consistent with values reported for identical systems. Under these conditions, n ≈ 1.7 and k ≈ 0 in the near‐infrared (NIR) to mid‐IR region (Figure S12). The optical behavior of each array was simulated (arrays shown in Figure 4A–C, see Figure S13A for exact habit dimensions used in the simulation). Due to the large periodicity between the habits (5 µm), initial simulation results (Figure S13B) reveal small peaks in the reflection spectra because of weak inter‐superlattice coupling. Despite this weak inter‐superlattice coupling, which reduces the intensity of the resonance peaks, the reflection spectra of the various superlattice habits differ. However, given their low amplitude, these peaks are not readily observed experimentally.

To verify the inter‐superlattice coupling uncovered in the simulation, we designed and fabricated an array of cylindrical metastable superlattices with a 2‐µm pitch spacing (Figure 5A) to enhance coupling strength. During fabrication, a higher agitation rate (950 rpm) was necessary to successfully form superlattices in microwells with reduced inter‐well spacings. At a moderate agitation rate (650 rpm), the resulting superlattices were faceted and did not conform to the microwell geometry (Figure S14). In this dense microwell arrangement, elevated surface tension at the array boundaries may create capillary barriers that suppress convective mass transport between the bulk solution and the liquid confined in each microwell. As a result, each microwell may act as an isolated microreactor, where diffusion dominates and superlattices have sufficient time to facet into a thermodynamically favored habit. When the agitation rate was increased to 950 rpm, metastable superlattices confined to the microwell geometry formed, likely because the capillary barriers were disrupted and convective exchange was restored. SEM images, including those taken at a 45° tilt, confirm successful fabrication of microwell‐templated colloidal crystals under these conditions (Figure 5B, Figure S15).

**FIGURE 5:**
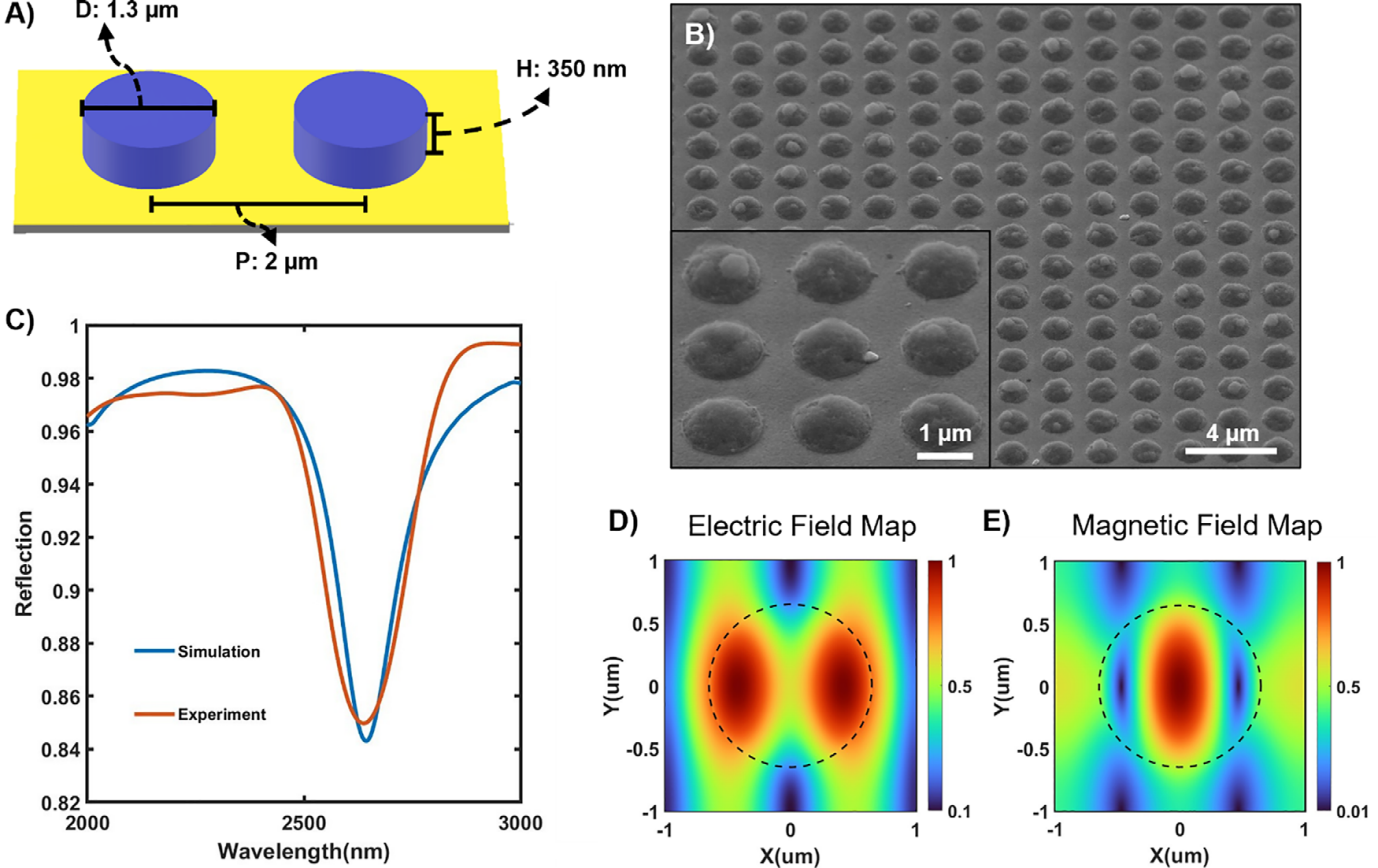
(A) Schematic of neighboring cylindrical metastable superlattices in a fabricated device, the device consists of a total of 900 superlattices. (B) 45° tilt‐view SEM images of a fabricated device. Large‐area SEM images are available in Figure S15. (C) Simulated and experimental reflection spectra. The simulated results are calculated using a matching index of 2.25. (D) Normalized electric field map and (E) normalized magnetic field map of cylindrical metastable superlattice resonator at the resonance in x‐y plane at z = 175 nm.

Reflection measurements were performed using a Fourier Transform Infrared (FTIR) spectroscopy instrument coupled to a microscope, and they revealed a stronger resonance peak arising from the Mie resonance at ≈2,600 nm (Figure 5C). However, the experimentally measured peak position does not match the simulated peak position (≈2,200 nm, Figure S16A), which was calculated using the refractive index from a perfect BCC lattice (Figure S12). This mismatch likely arises because the superlattices in the fabricated arrays may vary in habit height (e.g., overgrowth or undergrowth) and crystallinity; defects in the arrays could also play a role (Figure S15).

Post‐processing, in particular the silica embedding process, can also contribute to the mismatch in the peak position between the simulated and experimental systems. Silica embedding is known to contract the DNA in the superlattices, reducing the interparticle spacing in all directions. This densification in the superlattices can cause the effective refractive index to increase as compared to the calculated values (Figure S12). Indeed, the effective refractive index of a BCC lattice as a function of the percent contraction of the lattice was simulated (Figure S16B), and it was confirmed that lattice contraction increases the refractive index of superlattice up to ≈2.3 at a 30% lattice contraction. To investigate how the effective index affects the overall optical behavior of the system, we simulated the reflection spectra with effective indices of 2 and 2.25 while holding all other variables constant (Figure S16A). Excellent agreement between the experimental and simulated results was observed when an index of 2.25 was used in the simulation of the cylindrical habits (Figure 5C). This agreement suggests that densification‐induced lattice contraction is likely to contribute substantially to the mismatch observed between the experimental and preliminary simulation results, although we cannot rule out additional contributions from substrate coupling or finite‐size effects not captured in the idealized retrieval model. We note that the degree of lattice contraction was not measured directly but was inferred from the improved agreement between simulated and experimental spectra at the adjusted index. To better understand the nature of the optical modes supported by the resonators, electric and magnetic field distributions were simulated at the resonance wavelength. The simulated electric and magnetic field maps reveal a characteristic electric dipole mode aligned along the X‐axis (Figure 5D) as well as a centrally symmetric magnetic field representing a magnetic dipole mode aligned out of plane (along z) (Figure 5E), respectively. These results confirm that the metastable superlattices function as resonators that support magnetic Mie resonances in the designed array structure (Figure S17).

While this study focused on advancing habit control and device‐level design in colloidal crystal engineering with DNA, the broader framework is inherently modular. Parameters such as nanoparticle size and shape, core composition, DNA linker length, and overhang design, though held constant here, are readily tunable [8, 9, 38], and are known to influence the optical properties of superlattice resonators. Together, these degrees of freedom define a vast and versatile design space for engineering metasurfaces with tailored functionality. Future work will systematically explore these parameters to uncover array‐level optical trends. With sufficient data, this new approach may ultimately enable inverse design strategies powered by machine learning.

## 3 Conclusions

In conclusion, this work expands the capabilities of colloidal crystal engineering with DNA by establishing a highly modular, multi‐scale platform for constructing meta‐structures from metamaterial building blocks, in particular, nanoparticle superlattices with programmable structure and optical properties. By integrating DNA‐guided assembly with lithographically defined microwells, metastable superlattices with prescribed habits were reproducibly formed through kinetically driven crystallization. This decoupling of crystal habit from lattice symmetry unlocks a previously inaccessible design space for engineering non‐equilibrium habits with functional consequences. This strategy offers new opportunities for integrating colloidal superlattices into devices where superlattice habit and/or spatial control is critical. As a proof‐of‐concept, an optically functional metasurface composed of cylindrical superlattices was fabricated, exhibiting a Mie resonance peak in the near‐infrared, with experimental results closely aligning with the simulated results.

Taken together, these findings demonstrate a potentially scalable framework for tuning optical response through structural parameters across multiple hierarchical levels, from the nanoscale to the mesoscale. In particular, the ability to independently vary nanoparticle composition, DNA linker design, lattice symmetry, and superlattice habit provides a versatile route toward engineering optical responses not readily accessible through conventional top‐down fabrication approaches. This level of control provides a powerful foundation for the deliberate design of metamaterials, nanophotonic devices, and optical sensing platforms.

## 4 Methods

### 4.1 Materials

DNA synthesis reagents were purchased from Glen Research. Gold nanoparticles were purchased from Ted Pella. Triethoxysilane (TES), tris(2‐carboxyethyl)phosphine (TCEP), NaCl, NaH_2_PO_4_, Na_2_HPO_4_, and sodium dodecyl sulfate (SDS) were purchased from Sigma‐Aldrich. N‐Trimethoxysilylpropyl‐N,N,N‐trimethylammonium chloride (TMSPA) was purchased from Gelest.

### 4.2 DNA Synthesis

Oligonucleotides were synthesized at a 5 µmol scale on an automated MerMade 12 synthesizer (BioAutomation). Universal controlled pore glass (CPG) beads were used as the solid support for linker DNA strands, while 3′ thiol was used for anchor strands. Oligonucleotides were cleaved from the solid support according to manufacturer protocols and contained a 5′ dimethoxytrityl group to facilitate purification via reversed‐phase HPLC. Purified strands were treated with acid deprotection and extracted with ethyl acetate. Matrix‐assisted laser desorption ionization time‐of‐flight mass spectrometry (MALDI‐TOF, Bruker) was used to confirm the molecular weight of oligonucleotides, and UV−vis spectroscopy (Agilent) was used to quantify absorption so that concentration could be determined. Extinction coefficients were calculated for 260 nm using the OligoAnalyzer tool (Integrated DNA Technologies).

### 4.3 Superlattice Assembly

Superlattices were assembled in microwells by mixing the PAEs, patterned substrates, and linkers in a 5‐mL Eppendorf tube, and they were subjected to a previously established slow‐cooling protocol on a thermomixer (Eppendorf Thermomixer C Model 5382). Superlattices were transferred to the solid‐state by embedding them in silica and then they were visualized using an SEM (Hitachi SU‐8030/JEOL JMS‐7900FLV). Detailed methods can be found in the Supporting Information.

### 4.4 Optical Measurements

Reflection measurements were performed using a Fourier‐transform infrared (FTIR) setup (Hyperion Vertex 70) coupled with an optical microscope. An aperture size of approximately 30 × 30 µm^2^ was used to illuminate the sample. For the reflection measurements, an optically thick gold (Au) film was used as a reference.

## Conflicts of Interest

The authors declare no conflict of interest.

## Supporting information




**Supporting File**: smll75174‐sup‐0001‐SuppMat.docx.

## Data Availability

The data that support the findings of this study are available from the corresponding author upon reasonable request.

## References

[1] J. Andreo , R. Ettlinger , O. Zaremba , et al., “Reticular Nanoscience: Bottom‐Up Assembly Nanotechnology,” Journal of the American Chemical Society 144, no. 17 (2022): 7531–7550, 10.1021/jacs.1c11507. 35389641

[2] G. von Freymann , V. Kitaev , B. V. Lotsch , and G. A. Ozin , “Bottom‐up Assembly of Photonic Crystals,” Chemical Society Reviews 42, no. 7 (2013): 2528–2554. 10.1039/c2cs35309a 23120753

[3] W. Baek , H. Chang , M. S. Bootharaju , J. H. Kim , S. Park , and T. Hyeon , “Recent Advances and Prospects in Colloidal Nanomaterials,” JACS Au 1, no. 11 (2021): 1849–1859. 10.1021/jacsau.1c00339 PMC8611664 34841404

[4] A. Loiudice , O. Segura Lecina , and R. Buonsanti , “Atomic Control in Multicomponent Nanomaterials: When Colloidal Chemistry Meets Atomic Layer Deposition,” ACS Materials Letters 2, no. 9 (2020): 1182–1202, 10.1021/acsmaterialslett.0c00271.

[5] R. J. Macfarlane , B. Lee , M. R. Jones , N. Harris , G. C. Schatz , and C. A. Mirkin , “Nanoparticle Superlattice Engineering With DNA,” Science 334, no. 6053 (2011): 204–208, 10.1126/science.1210493. 21998382

[6] D. Nykypanchuk , M. M. Maye , D. van der Lelie , and O. Gang , “DNA‐guided Crystallization of Colloidal Nanoparticles,” Nature 451, no. 7178 (2008): 549–552, 10.1038/nature06560. 18235496

[7] R. J. Macfarlane , M. R. Jones , A. J. Senesi , et al., “Establishing the Design Rules for DNA‐Mediated Programmable Colloidal Crystallization,” Angewandte Chemie International Edition 49, no. 27 (2010): 4589–4592, 10.1002/anie.201000633. PMC3039301 20486143

[8] D. Samanta , W. Zhou , S. B. Ebrahimi , S. H. Petrosko , and C. A. Mirkin , “Programmable Matter: The Nanoparticle Atom and DNA Bond,” Advanced Materials 34, no. 12 (2022): 2107875, 10.1002/adma.202107875. 34870875

[9] C. R. Laramy , M. N. O'Brien , and C. A. Mirkin , “Crystal Engineering With DNA,” Nature Reviews Materials 4, no. 3 (2019): 201–224, 10.1038/s41578-019-0087-2.

[10] C. A. Mirkin and S. H. Petrosko , “Inspired beyond Nature: Three Decades of Spherical Nucleic Acids and Colloidal Crystal Engineering With DNA,” ACS Nano 17, no. 17 (2023): 16291–16307, 10.1021/acsnano.3c06564. 37584399

[11] R. J. Macfarlane , R. V. Thaner , K. A. Brown , et al., “Importance of the DNA “bond” in programmable nanoparticle crystallization,” Proceedings of the National Academy of Sciences 111, no. 42 (2014): 14995–15000, 10.1073/pnas.1416489111. PMC4210335 25298535

[12] X. Ye , J. Chen , B. T. Diroll , and C. B. Murray , “Tunable Plasmonic Coupling in Self‐Assembled Binary Nanocrystal Superlattices Studied by Correlated Optical Microspectrophotometry and Electron Microscopy,” Nano Letters 13, no. 3 (2013): 1291–1297, 10.1021/nl400052w. 23418862

[13] S. Kim , C. Y. Zheng , G. C. Schatz , K. Aydin , K.‐H. Kim , and C. A. Mirkin , “Mie‐Resonant Three‐Dimensional Metacrystals,” Nano Letters 20, no. 11 (2020): 8096–8101, 10.1021/acs.nanolett.0c03089. 33054221

[14] Y. Zhang , D. D. Xu , I. Tanriover , et al., “Nonlinear Optical Colloidal Metacrystals,” Nature Photonics 19 (2025): 20–27.

[15] M. B. Ross , J. C. Ku , V. M. Vaccarezza , G. C. Schatz , and C. A. Mirkin , “Nanoscale form Dictates Mesoscale Function in Plasmonic DNA–Nanoparticle Superlattices,” Nature Nanotechnology 10, no. 5 (2015): 453–458, 10.1038/nnano.2015.68. 25867942

[16] N. Kim , J.‐H. Huh , Y. Cho , et al., “Achieving Optical Refractive Index of 10‐Plus by Colloidal Self‐Assembly,” Small 20, no. 45 (2024): 2404223, 10.1002/smll.202404223. 39082408

[17] S. Lee , “Colloidal Superlattices for Unnaturally High‐Index Metamaterials at Broadband Optical Frequencies,” Optics Express 23, no. 22 (2015): 28170, 10.1364/OE.23.028170. 26561088

[18] E. Auyeung , T. I. N. G. Li , A. J. Senesi , et al., “DNA‐mediated Nanoparticle Crystallization Into Wulff Polyhedra,” Nature 505, no. 7481 (2014): 73–77, 10.1038/nature12739. 24284632

[19] M. N. O'Brien , H. X. Lin , M. Girard , et al., “Programming Colloidal Crystal Habit With Anisotropic Nanoparticle Building Blocks and DNA Bonds,” Journal of the American Chemical Society 138, no. 44 (2016): 14562–14565. 10.1021/jacs.6b09704 27792331

[20] S. Y. Park , A. K. R. Lytton‐Jean , B. Lee , S. Weigand , G. C. Schatz , and C. A. Mirkin , “DNA‐programmable Nanoparticle Crystallization,” Nature 451, no. 7178 (2008): 553–556, 10.1038/nature06508. 18235497

[21] M. Ye , T. Hueckel , P. P. Gatenil , K. Nagao , W. C. Carter , and R. J. Macfarlane , “Nanoparticle Superlattices With Nonequilibrium Crystal Shapes,” ACS Nano 18, no. 24 (2024): 15970–15977, 10.1021/acsnano.4c04192. 38838258

[22] S. E. Seo , M. Girard , M. Olvera de la Cruz , and C. A. Mirkin , “Non‐equilibrium Anisotropic Colloidal Single Crystal Growth With DNA,” Nature Communications 9, no. 1 (2018): 4558, 10.1038/s41467-018-06982-9. PMC6212572 30385762

[23] G. C. Sosso , J. Chen , S. J. Cox , et al., “Crystal Nucleation in Liquids: Open Questions and Future Challenges in Molecular Dynamics Simulations,” Chemical Reviews 116, no. 12 (2016): 7078–7116, 10.1021/acs.chemrev.5b00744. PMC4919765 27228560

[24] C. Jia , A. Xiao , J. Zhao , et al., “A New Perspective on Crystal Nucleation: A Classical View on Non‐Classical Nucleation,” Crystal Growth & Design 24, no. 1 (2024): 601–612, 10.1021/acs.cgd.3c01078.

[25] D. Erdemir , A. Y. Lee , and A. S. Myerson , “Nucleation of Crystals From Solution: Classical and Two‐Step Models,” Accounts of Chemical Research 42, no. 5 (2009): 621–629, 10.1021/ar800217x. 19402623

[26] T. Hueckel , D. J. Lewis , A. Mertiri , D. J. D. Carter , and R. J. Macfarlane , “Controlling Colloidal Crystal Nucleation and Growth With Photolithographically Defined Templates,” ACS Nano 17, no. 21 (2023): 22121–22128, 10.1021/acsnano.3c09401. 37921570

[27] A. M. Wong , K. Je , C. Y. Zheng , et al., “Arrays of Colloidal Single Crystals Engineered With DNA in Lithographically Defined Microwells,” Nano Letters 23, no. 1 (2023): 116–123, 10.1021/acs.nanolett.2c03713. 36541890

[28] C. Y. Zheng , Y. Yao , J. Deng , et al., “Confined Growth of DNA‐Assembled Superlattice Films,” ACS Nano 16, no. 3 (2022): 4813–4822, 10.1021/acsnano.2c00161. 35213130

[29] S. Karthika , T. K. Radhakrishnan , and P. Kalaichelvi , “A Review of Classical and Nonclassical Nucleation Theories,” Crystal Growth & Design 16, no. 11 (2016): 6663–6681, 10.1021/acs.cgd.6b00794.

[30] E. Auyeung , R. J. Macfarlane , C. H. J. Choi , J. I. Cutler , and C. A. Mirkin , “Transitioning DNA‐Engineered Nanoparticle Superlattices From Solution to the Solid State,” Advanced Materials 24, no. 38 (2012): 5181–5186, 10.1002/adma.201202069. 22810947

[31] K. F. Kelton and A. L. Greer , “The Classical Theory,” in Pergamon Materials Series (2010), 19–54.

[32] C. N. Nanev , “Kinetics and Intimate Mechanism of Protein Crystal Nucleation,” Progress in Crystal Growth and Characterization of Materials 59, no. 4 (2013): 133–169, 10.1016/j.pcrysgrow.2013.09.001.

[33] M. Sleutel and A. E. S. Van Driessche , “Role of Clusters in Nonclassical Nucleation and Growth of Protein Crystals,” Proceedings of the National Academy of Sciences 111, no. 5 (2014): E546–E553, 10.1073/pnas.1309320111. PMC3918807 24449867

[34] O. Gliko , N. Neumaier , W. Pan , et al., “A Metastable Prerequisite for the Growth of Lumazine Synthase Crystals,” Journal of the American Chemical Society 127, no. 10 (2005): 3433–3438, 10.1021/ja043218k. 15755162

[35] F. C. Meldrum and H. Cölfen , “Controlling Mineral Morphologies and Structures in Biological and Synthetic Systems,” Chemical Reviews 108, no. 11 (2008): 4332–4432, 10.1021/cr8002856. 19006397

[36] J. Aizenberg , “New Nanofabrication Strategies: Inspired by Biomineralization,” MRS Bulletin 35, no. 4 (2010): 323–330, 10.1557/mrs2010.555.

[37] F. C. Meldrum and C. O'Shaughnessy , “Crystallization in Confinement,” Advanced Materials 32, no. 31 (2020): 2001068, 10.1002/adma.202001068. 32583495

[38] M. R. Jones , N. C. Seeman , and C. A. Mirkin , “Programmable Materials and the Nature of the DNA Bond,” Science 347, no. 6224 (2015): 1260901, 10.1126/science.1260901. 25700524

