# Supplementary material for: Multi‐Scale Engineering of Photonic Meta‐Structures With Emergent Optical Resonances via Colloidal Crystal Engineering With DNA and Microwell Templates
Source: Small. 2026 Aug 12;22(58):e75174. doi: 10.1002/smll.75174 (PMC13676837; doi:10.1002/smll.75174)
Supplement: Supplementary file 1 [file SMLL-22-e75174-s001.docx]

**Supporting Information**

**Multi-Scale Engineering of Photonic Meta-structures with Emergent Optical Resonances via Colloidal Crystal Engineering with DNA and Microwell Templates**
Alexa M. Wong^1,2^, Ramin Yazdaanpanah^2,4^, Mia J. P. Pascall^1,2^, Jeongmin Cho^2,3^, Yiming Yang^1,2^, Allen X. Guo^2,3^, Janice Kang^1,2^, Koray Aydin^2,4^*, and Chad A. Mirkin^1,2,3^*

^1^ Department of Chemistry, Northwestern University, Evanston, IL, USA

^2^ International Institute for Nanotechnology, Northwestern University, Evanston, IL, USA

^3^ Department of Materials Science and Engineering, Northwestern University, Evanston, IL, USA

^4^ Department of Electrical and Computer Engineering, Northwestern University, Evanston, IL, USA.

**Table of Contents:**

| 1. Materials and Methods | 3 |
| --- | --- |
| a) Surface template fabrication | 3 |
| b) Nanoparticle and substrate DNA functionalization | 3–4 |
| c) PAE assembly and cooling protocol | 4 |
| d) Silica embedding protocol | 4 |
| 2. Effect of prolonged silica incubation time on PMMA template removal | 5 |
| 3. Additional SEM images of samples from varying supersaturation conditions | 6-8 |
| 4. UV-vis spectroscopy of recovered PAEs from homogeneous crystallization pathways | 9–11 |
| 5. Large-area SEM image of superlattices with habit evolution | 12-14 |
| 6. Effect of annealing on kinetically-trapped superlattices | 15 |
| 7. Optical simulation methods | 16-17 |
| 8. Simulated optical properties of metastable superlattice arrays (5 µm spacing) | 18 |
| 9. Effect of agitation on superlattice arrays with reduced spacing | 19 |
| 10. Large-area SEM images of fabricated metasurfaces | 20 |
| 11. Effect of lattice contraction and reflection spectra with different refractive indices | 21-22 |
| 12. Mie Resonance Behavior | 23 |
| 13. References | 24 |

1. **Materials and methods**
a. **Surface template fabrication**

Gold-coated Si wafers were prepared via e-beam evaporation (2-nm Cr adhesion layer and 100-nm Au layer). The pre-cut, 10 mm x 10 mm gold coated wafers were solvent-cleaned with acetone and isopropanol. They were then cleaned via an O_2_ plasma at 50 W for 2 min. After that, a layer of polymethyl methacrylate (PMMA) was spin-coated onto the 10 mm x 10 mm gold-coated wafers and subsequently annealed on a hot plate at 180 ºC (See **Table S1** for spin coating conditions and PMMA used) ^[1]^. E-beam lithography (FEI Quanta 650 ESEM) was performed on the substrates; 4 arrays of templates were on each 10 mm x 10 mm substrate. Each microwell is 1 µm × 1 µm × 1 µm in volume. They were then developed in positive radiation resist developer (MBIK/IPA 1:3) for 2 min with gentle agitation, rinsed with IPA, and dried under a N_2_ stream. Each substrate was then cut into four 5 mm x 5 mm substrates for DNA functionalization. For experiments in Figure 1 and Figure 2, substrates with 50 x 100 arrays (5000 microwells) were used. For the remaining experiments, substrates with 20 x 20 arrays (400 microwells) were used.

| *PMMA* | *Spin rate (rpm)* | *Film thickness (nm)* |
| --- | --- | --- |
| 950 C7 | 4000 | 1000 |

**Table S1.** PMMA used and the associated spin coating conditions.

b. **Nanoparticle and substrate DNA functionalization**

All nanoparticles were purchased from Ted Pella. The DNA sequences used in this work were taken from the literature (**Table S2**) ^[2]^. Specifically, we functionalized the NPs and substrates with 3’ thiolated anchor DNA strands. Then, the complementary linker strands were hybridized onto the anchor strands functionalized on the NPs or substrates.

| *Description* | *DNA sequence (5’ to 3’)* |
| --- | --- |
| Anchor DNA A | TCA ACT ATT CCT ACC TAC (sp18)_2_ SH |
| Anchor DNA B | TCC ACT ATT CCT ACC TAC (sp18)_2_ SH |
| Complementary linker DNA A | GTA GGT AGG AAT AGT TGA S TTCCTT |
| Complementary linker DNA B | TTG CTG AGT ATG AGT GGA S AAGGAA |

**Table S2.** DNA Sequences used in this work. “sp18” refers to a hexaethylene glycol spacer unit manufactured by Glen Research.

NPs were functionalized with anchor DNA using a slightly modified procedure described by Sun et al. ^[4]^ Briefly, 3’ alkylthiol-modified oligonucleotides were treated with a 200 mM solution of tris(2-carboxyethyl)phosphine (TCEP), and then purified through a 3k ultra centrifugal filter (Amicon ®) at least 5 times to remove the excess TCEP. The oligonucleotides are then added into the citrate-capped AuNP solution, the NP solution was then brought to 0.01 M sodium phosphate buffer (pH = 7.4) and 0.01 wt. % sodium dodecyl sulfate (SDS) in water. Salt-aging of the NPs was performed immediately to bring to the NaCl concentration to 0.05M, then every 30 min, the NaCl concentration was increased to 0.1 M, 0.2 M, and 0.3M, respectively. After salt-aging, the NPs solution was left overnight with gentle agitation. The NP solution was then centrifuged in 50k spin filters (MilliporeSigma) three times with water to remove the excess DNA. In between each centrifugation, the NPs were resuspended in 0.5 M NaCl, 0.01 M sodium phosphate buffer (pH = 7.4) and 0.01 wt. % SDS solution.

The templated substrates were functionalized with anchor DNA in a similar fashion. After the DNA was treated with TCEP and purified, it was added to a 0.5 M NaCl PBS solution with the substrates, then left on a shaker at 650 rpm overnight.

c. **PAE assembly and cooling protocol**

After functionalizing the substrates with A or B anchor DNA, the respective linkers were hybridized onto the anchor DNA. Specifically, the substrates were rinsed with 0.5 M NaCl PBS and put into a 1.5-mL Eppendorf tube containing the linkers in 0.5 M NaCl PBS. They were then put in a shaker at 55 ºC and 200 rpm for 30 min. After 30 min, the Eppendorf tubes were taken out and cooled to room temperature. The substrates could then be placed into PAE solutions in an upright orientation that contained the functionalized PAEs and linkers for superlattice growth. The mixture of PAE solution and substrate were placed in a shaker. The samples were subjected to an identical cooling protocol that started with a 10h annealing period, followed by 1 ºC drop, followed by a 10h annealing period, and so forth. A good rule of thumb is to start the cooling protocol at least 6 ºC above the T_m_ of the PAEs (e.g. T_m_ = 39 ºC, start at 45 ºC), then cooled until PAEs in the solution were depleted. All experiments except for device fabrication were performed under 650 rpm.

d. **Silica embedding**

The silica embedding procedure was slightly modified from a procedure taken from the literature. The substrates were transferred to 1.5-mL Eppendorf tubes with 500 µL of 0.5 M NaCl PBS solution. Two μL of N, trimethoxysilylpropyl-N,N,Ntrimethylammonium chloride (TMSPA) was added to the tube, and it was left on a shaker at 950 rpm for 20 min. Then, 5 μL of triethoxysilane (TES) was added to the solution, and the mixture was left on the shaker for another hour so that a silica network formed around the superlattices and transfer them into solid state. Note that if TES was left in the solution for longer than an hour, silica network would start to form in the PMMA around the superlattices, making the PMMA impossible to remove. Lastly, the substrate was removed from the solution, rinsed with water, and dried under a stream of N_2_.

2. **Effect of prolonged silica incubation time on PMMA template removal**

**Figure S1.** PAE superlattice array transferred to the solid state along with the PMMA template under previously established protocol (20 min with N-trimethoxysilylpropyl-N,N,N-trimethylammonium chloride, followed by overnight treatment with triethoxysilane).

3. **Additional SEM images of samples from varying supersaturation conditions**

**Figure S2.** Additional SEM images of PAE architectures grown in high supersaturation conditions (10 OD). Scale bars = 200 nm.

**Figure S3.** Additional SEM images of PAE architectures grown in moderate supersaturation condition (5 OD). Scale bars = 200 nm.

**Figure S4.** Additional SEM images of PAE architectures grown in low supersaturation condition (2.4 OD). Scale bars = 200 nm.

4. **UV-vis spectroscopy of recovered PAEs from homogeneous crystallization pathways**

**Figure S5.** UV-vis spectroscopy of recovered PAEs from supernatant under high supersaturation condition, dilution factor = 10.

**Figure S6.** UV-vis spectroscopy of recovered PAEs from supernatant under moderate supersaturation condition, dilution factor = 3.

**Figure S7.** UV-vis spectroscopy of recovered PAEs from supernatant under low supersaturation condition, dilution factor = 1.

5. **Large-area SEM image of superlattices with habit evolution**

**Figure S8.** Larger-area SEM image of a portion of the superlattice array, where the superlattices were deeply seated in the microwells at low PAE loading conditions. The structures did not exhibit faceting. Scale bar: 2µm.

**Figure S9.** Larger-area SEM image of a portion of the superlattice array, where the superlattices were seated in the microwells at moderate PAE loading conditions. The structures had yet to interface bulk liquid and did not exhibit faceting. Scale bar: 2µm.

**Figure S10.** Larger-area SEM image of a portion of the superlattice array, where the superlattices were approaching the top of the microwells at high PAE loading conditions. All superlattices exhibit faceting and thermodynamic preferred morphology. Scale bar: 2µm.

6. **Effect of Annealing on Kinetically-trapped Superlattices**

**Figure S11.** Evolution of superlattice in microwell after annealing. While some ordering occurred during the annealing step, the superlattices were unable to facet with additional annealing time. **(A)** SEM image of superlattice right after the slow cooling process, which completed at 35˚C. **(B)** SEM image of superlattice subjected to a 10 h annealing step at 35˚C. Scale bars: 500 nm.

7. **Optical simulation methods**

**Figure S12.** Calculated complex refractive index value of a perfect BCC lattice constructing from 20 nm gold nanoparticles with 40 nm lattice parameter.

We employed Lumerical, a commercial Maxwell solver based on the finite-difference time-domain (FDTD) method, to compute the scattering matrix of the BCC structure containing 20 nm nanoparticles while the lattice parameter is 40 nm. Following the extraction of complex scattering parameters (S-parameters) from the electromagnetic simulations, the effective refractive index was determined through an iterative retrieval process as described below:

$$n_{eff}=\frac{1}{kd}\cos^{-1} \left( \frac{1-S_{11}^{2}+S_{21}^{2}}{2S_{21}} \right)$$

Here, *k* and *d* represent the wavevector and the thickness of the structure, respectively^[3]^. In this formulation, the system is assumed to be symmetric along the direction of light propagation ([100] direction), resulting in the conditions that S_11_ = S_22_ and S_21_ = S_12_ . For the material properties, we used the Johnson and Christy dataset for the refractive index of gold nanoparticles^4^. The lattice constant of the structure was extracted from experimental data (40 nm).

To accurately determine the effective refractive index, the crystals were modeled as infinitely periodic in the plane perpendicular to the direction of light propagation (x–y plane). Additionally, increasing the number of unit cells along the z-direction (light propagation axis) improves the accuracy of the effective index calculation. Based on this, we used 10 unit cell layers in the z-direction for both BCC and FCC structures, which strikes a balance between computational cost and accuracy.

In the simulations, periodic boundary conditions were applied in the x- and y-directions, while perfectly matched layers (PML) were used along the z-direction to eliminate artificial reflections. All simulations were performed with maximum mesh accuracy, with a minimum mesh size of 0.25 nm to ensure numerical precision.

Simulations related to calculate the reflection of the cylindrical, triangular and ring shape resonators were also performed using Lumerical software. Here, we used an override mesh with a constant 5nm mesh size in all directions around the habit to increase accuracy. Each of these habits were considered to be placed on 100 nm gold layer on the top of silicon wafer. The periodic boundary conditions applied in x and y directions and a perfectly matched layer (PML) in z-direction.

8. **Simulated optical properties of metastable superlattice arrays (5 µm spacing)**

**Figure S13. (A)** Dimensions of the cylindrical, ring-like, and triangular prism habits used in simulations. **(B)** Simulated reflection spectra of habit arrays with 5 µm pitch spacing.

9.**Effect of agitation on superlattice arrays with reduced space**

**Figure S14.** Tilt view 45º SEM images of device fabricated under an agitation rate of **(A)** 650 rpm and **(B)** 950 rpm.

10. **Large-area SEM images of fabricated metasurfaces**

**Figure S15. (A)** Top-view large-area SEM image and **(B)** tilt-view (45º) large-area SEM image of fabricates metasurfaces.

11. **Effect of lattice contraction and reflection spectra with different refractive indices**

**Figure S16. (A)** Reflection spectra of arrays consisting of cylindrical metastable superlattice with a range of effective refractive indices and the experimental reflection spectrum. **(B)** Effective refractive index of the perfect BCC lattice from **Figure S9A** as a function of % contraction in the lattice.

To understand how index change affects the optical behavior of cylindrical superlattice arrays, we simulated the reflection spectra of these arrays while sweeping the refractive index of the cylindrical habit, as shown in **Figure S16A**. The results illustrate that as the refractive index increases from its original value (n=1.7 and k~0 in mid-IR regime for a perfect BCC superlattice with 20 nm nanoparticles and lattice constant of 40 nm) due to the factors such as contraction, the resonance wavelength shifts toward longer wavelengths, and the resonance intensity decreases. When comparing these simulations to experimental data (also shown in Figure S13A), there is good agreement between experiment and simulation when assuming an effective index between 2.25 for cylindrical habits. Therefore, the combined effects of lattice contraction, polycrystallinity, and defects can collectively shift the effective refractive index of cylindrical habits from 1.7 (calculated effective index of BCC lattice in figure S9) to approximately ~2.25.

**Figure S16B** shows the real part of the effective refractive index (n) of the superlattices in the mid-IR range as a function of lattice contraction. The method used to calculate the effective refractive index is described in Section S6. As shown in Figure S9, the refractive index remains nearly constant across the mid-IR spectrum, and the extinction coefficient is close to zero. Therefore, in Figure S13B, we focus only on the real index values in the mid-IR regime (at a wavelength of ~2500 nm). As observed in Figure S13B, for a BCC superlattice made of 20 nm gold nanoparticles, as the lattice contracts from the original 40 nm, the refractive index increases in a nonlinear fashion. This index increase continues such that with a 33% lattice contraction, the refractive index rises by 50%, from its original value of 1.7 to 2.55. It is worth mentioning that lattice contraction of more than 35 % will increase extinction coefficient (k) and the superlattices will no longer act as a dielectric in the mid-IR regime.

12. Mie Resonance Behavior

Figure S17 demonstrates the reflection spectra of cylindrical superlattice resonators with varying diameters while keeping the remaining structural parameters fixed. As shown in this figure, the resonance systematically redshifts with increasing resonator diameter, consistent with the expected scaling behavior of Mie-type cavity resonances.

In addition to the size-dependent spectral evolution, the simulated electric and magnetic near-field distributions continue to exhibit the characteristic circulating displacement current and enhanced magnetic field confinement associated with magnetic dipole Mie modes.

**Figure S17.** Diameter–wavelength reflectance map: simulated reflection spectra of cylindrical superlattice resonators with varying diameters while keeping the remaining structural parameters fixed.

**References**

1. NANO™ PMMA and Copolymer, MicroChem Corp, 2001, https://kayakuam.com/wpcontent/uploads/2019/09/PMMA_Data_Sheet.pdf. Accessed 12 March 2020.

2.	Landy, K. M.; Gibson, K. J.; Urbach, Z. J.; Park, S. S.; Roth, E. W.; Weigand, S.; Mirkin, C. A., Programming “Atomic Substitution” in Alloy Colloidal Crystals Using DNA. Nano Letters 2022, 22 (1), 280-285.

3.	Smith, D. R.; Vier, D. C.; Koschny, T.; Soukoulis, C. M. Electromagnetic Parameter Retrieval from Inhomogeneous Metamaterials. *Phys Rev E Stat Nonlin Soft Matter Phys* 2005, *71* (3). <https://doi.org/10.1103/PHYSREVE.71.036617/FIGURES/7/MEDIUM>.

4.	Johnson, P. B.; Christy, R. W. Optical Constants of the Noble Metals. *Phys Rev B* 1972, *6* (12), 4370–4379. <https://doi.org/10.1103/PhysRevB.6.4370>.
